# Andrographolide Suppresses Expressions of Coagulation and Fibrinolytic Inhibition-Related Factors in LPS-Induced Alveolar Epithelial Cell Type II via NF-κB Signal Pathway In Vitro

**DOI:** 10.1007/s44231-022-00010-7

**Published:** 2022-07-13

**Authors:** Guixia Yang, Xiang Li, Qing Li, Chuan Xiao, Hong Qian, Huilin Yang, Feng Shen

**Affiliations:** 1 Department of Intensive Care Unit, Guizhou Medical University Affiliated Hospital, Guiyang, 550001 China; 2 Department of Intensive Care Unit, Guizhou Maotai Hospital, Zunyi, 564500 China; 3 Department of Intensive Care Unit, The Affiliated Sixth People’s Hospital of Shanghai Jiaotong University, Shanghai, 200233 China; 4 Department of Intensive Care Unit, The Second People’s Hospital of Guiyang, Guiyang, 550001 China

**Keywords:** Acute respiratory distress syndrome, Alveolar epithelial cell type 域, NF-κB, Andrographolide, Hypercoagulation, Fibrinolytic inhibition

## Abstract

**Background:**

Andrographolide (Andro) has been confirmed to ameliorate alveolar hypercoagulation and fibrinolysis inhibition via NF-κB pathway in acute respiratory distress syndrome (ARDS), but the specific target of Andro is unknown.

**Purpose:**

Our aim is to explore the specific target of Andro through which the drug exerted its effects on alveolar hypercoagulation and fibrinolytic inhibition in LPS-induced ARDS.

**Methods:**

AECII was treated with different doses of Andro for 1 h, and then stimulated with LPS for 24 h. Expressions of tissue factor (TF), plasminogen activator inhibitor (PAI)-1 and tissue factor pathway inhibitor (TFPI) were detected. Concentrations of thrombin-antithrombin complex (TAT), pro-collagen type III peptide (PIIIP), antithrombin III (ATIII) and activated protein C (APC) in cell supernatant were measured by enzyme linked immunosorbent assay (ELISA). NF-κB signaling pathways activation was simultaneously determined. AECII with p65 down-/over-expression were used as control.

**Results:**

Andro effectively inhibited TF and PAI-1 and promoted TFPI expressions on AECII induced by LPS stimulation. Andro also significantly suppressed the productions of TAT and PIIIP but promoted ATIII and APC secretions from the LPS-treated cell. Furthermore, Andro application obviously inhibited NF-κB signaling pathway activation provoked by LPS, as shown by decreased level of phosphorylation (p‑)-IKKβ/IKKβ, p-p65/p65 and p65 DNA binding activity. The effects of Andro on those factors were obviously strengthened by down- but were weakened by up-regulation of p65 gene in AECII cell.

**Conclusions:**

Our data demonstrates that targeting AECII is the mechanism by which Andro ameliorates alveolar hypercoagulaiton and fibrinolytic inhibition via NF-κB pathway in ARDS. Andro is worth to be clinically further studied in ARDS treatment.

## Introduction

Acute respiratory distress syndrome (ARDS), a common cause of death in intensive care units [1, 2], is a devastating clinical syndrome characterized by non-cardiogenic pulmonary edema, respiratory distress and hypoxemia [3–5]. Although some progresses were made in the treatment in ARDS, its mortality is still as high as 35–45% [6]. The complex pathogenesis of ARDS is mainly responsible for the refractoriness and high mortality in this disease. Our previous studies have shown that LPS-induced ARDS exhibited alveolar hypercoagulability and fibrinolytic inhibition [7], which are important characteristics of this disease [8]. Alveolar hypercoagulation and fibrinolytic inhibition causes a large number of microthrombosis in the pulmonary blood vessels, massive fibrin deposition in alveolar cavity, lung tissue fibrosis, etc., which are associated with reduced lung compliance, V/Q mismatch and with refractory hypoxia in ARDS [9]. Therefore, it is pivotal to explore effective drugs for alveolar hypercoagulation and fibrinolytic inhibition in this disease.

Our previous studies confirmed that NF-κB pathway participates the regulation of alveolar coagulation and fibrinolysis inhibition in ARDS, and alveolar epithelial cell type 域 (AEC域) was testified to be a main effector cell responsible for these pathophysiologies [10–12]. Therefore, targeting AEC域 cell could be efficacious for ARDS treatment.

Andrographolide (Andro) is the main active components of the natural plant andrographis paniculata. It owns many pharmacological effects, including anti-inflammation, antibacterial, anti-virus, liver protection and anti-vascular properties [13–17]. In our previous animal study, Andro was confirmed to effectively ameliorate alveolar hypercoagulation and fibrinolytic inhibition via NF-κB signal pathway [18], but the specific target cell of Andro is unknown. Given that AEC域cell has important regulatory role in alveolar hypercoagulation and fibrinolytic inhibition in ARDS, so we observed the efficacies of Andro on expressions of coagulation and fibrinolytic inhibition-related factors in AECII cell under LPS stimulation.

## Materials and Methods

### Cell Culture

The RLE‑6TN cell line (ACE II cell line from rats) were obtained from the Cell Bank of Xiangya Medical College. The cells were cultured in RPIM 1640 medium (Gibco; USA), supplemented with 10% FBS (Gibco; USA), penicillin (100 units/ml) (Hyclone; Cytiva) and streptomycin (100 μg/ml) (Hyclone; Cytiva) at 37 °C and 5% CO_2_.

### Detection of Andro Cytotoxicity by Using Cell Counting Kit-8 (CCK-8)

In order to determine the optimal concentration of Andro for RLE-6TN cells, we used CCK8 to detect Andro cytotoxicity. RLE-6TN cells were seeded into a 96-well plate (5 × 10^3^ cells/well in 100 μl volume) and pre-incubated for 24 h at 37 °C with 5% CO_2_. Different concentrations of Andro (0, 3.125, 6.25, 12.5, 25, 50, 100 µg/ml) were added into the wells. PBS was used as a blank control. The cells were cultured for 24 h in the incubator, and CCK-8 reagent (Dojindo Molecular Technologies, Inc.) was added at 10 μl/well for 1 h. The absorbance was measured at a wavelength of 450 nm using a microplate reader.

### Experimental Protocol

**Fig. 1:**
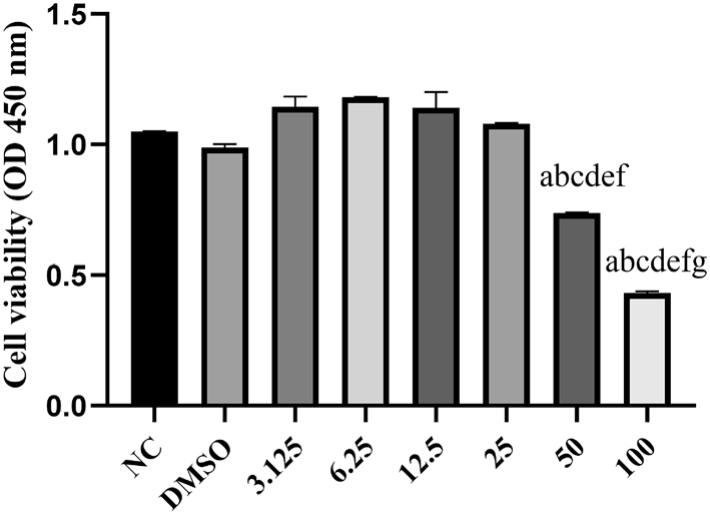
CCK8 was used to detect the cytotoxicity of Andro on RLE-6TN cells. Cells were treated with various concentrations of Andro ranging from 3.125 to 100 mg/l. Cell viability showed a gradual cell death when the drug dose exceeded 25 mg/l; therefore, the Andro concentration selected for this experiment was 6.25, 12.5, 25 mg/l. *Andro* andrographolide

According to the results of Andor cytotoxicity (Fig. 1), three different dosages of Andro, i.e., 6.25 µg/ml, 12.5 µg/ml and 25 µg/ml, were selected for the subsequent experiment. The cells were divided into five groups, LPS, Andro (subdivided into 6.25 µg/ml, 12.5 µg/ml and 25 µg/ml groups) and control (CN). In LPS group, cells were stimulated with 50 µg/ml LPS for 24 h, while the cells in Andro groups were co-cultured with different concentrations of Andro (Sigma, USA) for 1 h first and then were stimulated with a same dosage of LPS for another 24 h. There were no any manipulations for cells in control group (CN).

### Down-(p65^−/−^) and Over-(p65^+/+^) Expressions of p65 Gene Construction

In order to explore whether the mechanism of Andro is associated with NF-κB pathway, we constructed different levels of p65 expressions in the cell. Based on the p65 gene (Gene ID: 309165), we made cell lines with down- and over-expressions of p65 gene respectively by using the lentivirus transfection according to our previous method [11]. And then these cells were also treated by LPS with or without Andro administration.

### Reverse Transcription-Quantitative (RT-q) PCR

The mRNA expressions of TF, PAI-1 and TFPI were detected by RT-qPCR. GAPDH was used as an internal reference. Briefly, cells were collected, and total RNA was extracted using TRIzol^®^ reagent (Takara Bio, Japan). RNA concentration was assessed using a NanoDrop™ 2000 spectrophotometer (Thermo Fisher Scientific, Inc.). The A260/A280 ratio of the extracted RNA was adjusted to 1.8–2.0. The primer sequences used were as follows:

TF: 5′‑AAT GGG CAG ATA GAG TGT‑3′
  5′‑TCT GAT TGT GGG TTT GTA‑3′;
PAI‑1: 5′‑ACC AAC TTC GGA GTA AAA‑3′
  5′‑TTG AAT CCC ATA GCA TCT‑3′
TFPI: 5′‑AAA CTG AAG AAA GAC CAC GCC‑3′
  5′‑TGT ATC ATC GTC TTC CTC GGG‑3′;
GADPH: 5′‑CAA GTT CAA CGG CAC AG‑3′
  5′‑CCA GTA GAC TCC ACG ACA T‑3′;

The reactions were set up as follows:

5 μl SYBR Green mix (Takara Bio, Japan), 0.5 μl forward primer, 0.5 μl reverse primer, 1 μl cDNA template and 2.8 μl ddH_2_O, for a total reaction volume of 10 μl. The entire reaction system was pre-heated at 95 °C for 30 s. qPCR was then performed using the folling thermo-cycling procedure: 95 °C for 5 s, 60 °C for 34 s and 95 °C for 15 min, 60 °C for 1 min, 95 °C for 15 s for 40 cycles. After that, dissolution and amplification curves of the target gene were recorded following gene amplification. Specificity of the reaction was evaluated, and the Ct value was calculated according to the dissolution and amplification curve. Expressions of target genes were calculated using the 2^−ΔΔCt^ method, where ΔΔCt = (Ct, sample target-Ct, sample GAPDH)—(Ct, control target—Ct, control GAPDH).

### Western Blotting

The levels of p65, phosphorylated (p)-p65, IKKβ, p-IKKβ, TF, PAI-1and TFPI were determined by western blot analysis. After 24 h of LPS stimulation, the cells were washed with cold PBS. Total protein was extracted using RIPA buffer (Hunan Fenghui Biotechnology, China). Protein concentrations were measured with a BCA assay kit according to the manufacturer’s instructions. 10 μg of protein from each sample was resolved on 12% Tris–glycine gel using SDS-PAGE. Then protein bands were blotted into nitrocellulose membranes and incubated in blocking solution for 1 h followed by 24 h of incubation with antibodies targeting p65 (1:1000; CST, USA), p‑p65 (1:1000; CST, USA), IKKβ (1:1000; CST, USA), p‑IKKβ (1:1000; CST, USA), TF (1:1000; Abcam, UK), PAI-1 (1:1000; Abcam, UK), PAI‑1 (1:1000; Abcam, UK) at 4 °C. The secondary antibody (horseradish peroxidase-conjugated goat ant-rabbit immuno-globulin; 1:5000; Wuhan Sanying Biotechnology Co., Ltd, China) was added and incubated with horseradish blocking solution for 1 h at room temperature using the membrane chemiluminescence detection system (EMD Millipore). Relative band densities were quantified using Image J software 1.4.3 (National Institutes of Health).

### ELISA Assay

Cell supernatants and nuclear extract were harvested and stored at − 80 °C. Thrombin antithrombin (TAT), procollagen III propeptide (PIIIP), antithrombin III (ATIII), activated protein C (APC) (Shanghai Fanke Industrial Co., Ltd, China) and p65 DNA binding (Cayman Chemical, USA) were determined by using test kits according to the manufacturers protocol.

### Statistical Analysis

Data are presented as the mean ± SEM. Statistical significance was determined using one-way ANOVA followed by Tukey’s post hoc test. *p* < 0.05 was considered to be statistically significant.

## Results

### Andro Inhibits TF and PAI-1, but Promotes TFPI mRNA and Protein Expressions in LPS-Stimulated RLE-6TN Cells

**Fig. 2:**
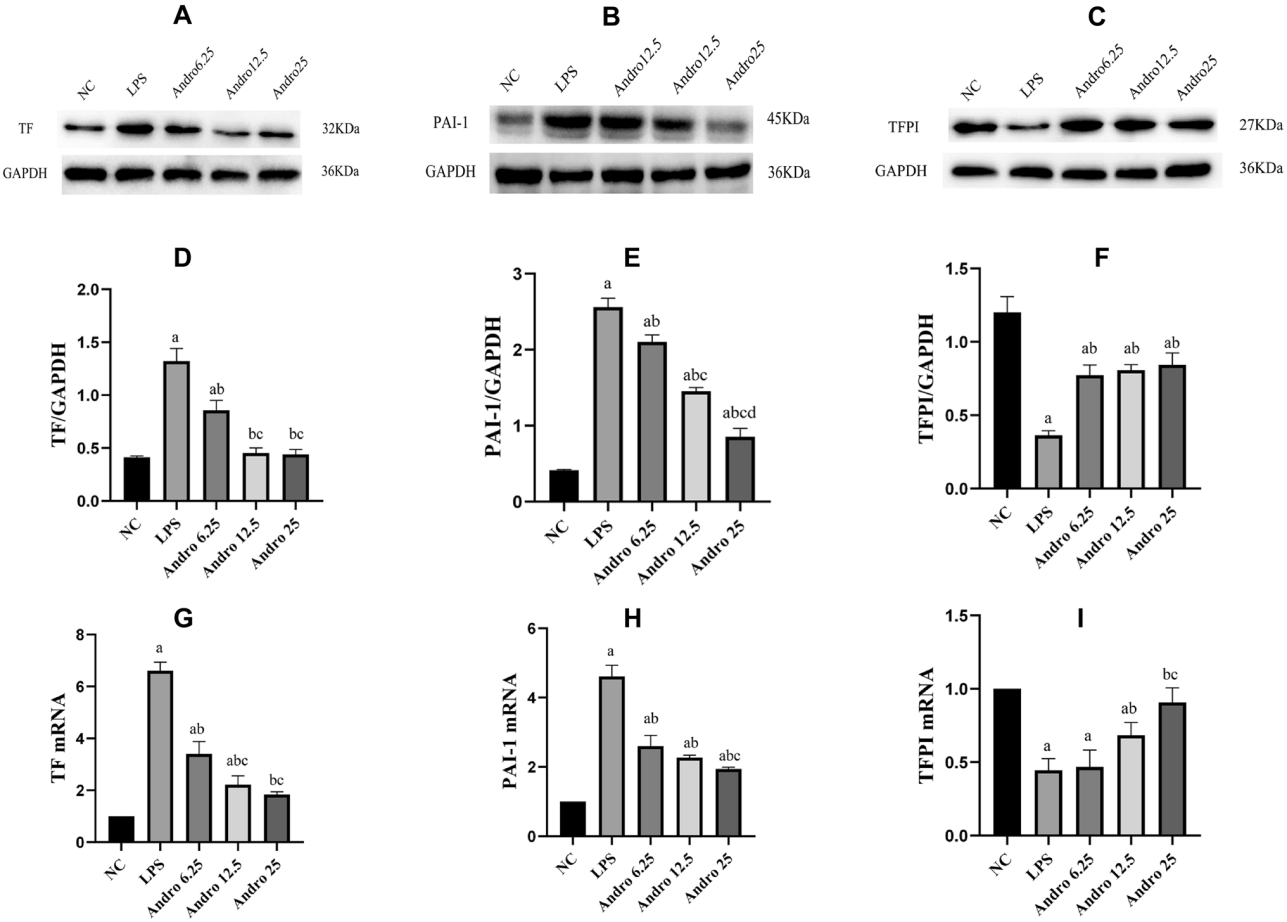
Effects of Andro on protein and mRNA expressions of TF, PAI-1 and TFPI induced by LPS in RLE-6TN cells. Protein and mRNA expressions of TF, PAI-1 and TFPI in the cells were measured by western-blotting and RT-PCR respectively. GAPDH was used as an internal control for protein reference in WB. Compared with group NC, a *p* < 0.05; Compared with group LPS, b *p* < 0.05; Compared with group Andro 6.25, c *p* < 0.05; Compared with group Andro 12.5, d *p* < 0.05. **A** and **D** the protein expression of TF. **B** and **E** the protein expression of PAI-1. **C** and **F** protein expression of TFPI. **G** TF mRNA expression. **I** PAI-1 mRNA expression. **J** TFPI mRNA expression. *TF* tissue factor, *TFPI* tissue factor pathway inhibitor, *PAI-1* plasminogen activator inhibitor-1, *WB* western blot, *(RT-q) PCR* Reverse transcription-quantitative, *Andro* andrographolide

LPS stimulation promoted TF, PAI-1, but inhibited TFPI expressions either in mRNA or in protein level in RLE-6TN cells, which were all reversed by Andro in dose-dependent manner. (Fig. 2).

### Andro Inhibits TAT and PIIIP but Promotes ATIII and APC Secretions from LPS Stimulated RLE-6TN Cells

**Fig. 3:**
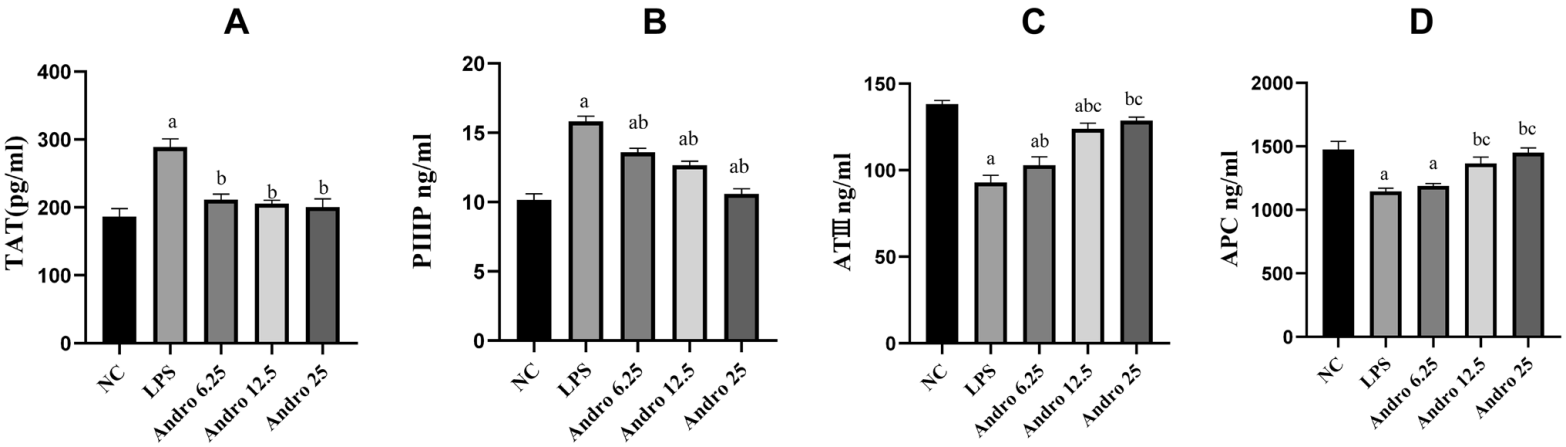
Effects of Andro on the secretions of PIIIP, TAT, ATIII and APC from RLE-6TN cells induced by LPS. ELISA was performed to measure the concentrations of PIIIP, TAT, ATIII and APC in supernatants. Compared with group NC, a *p* < 0.05; Compared with group LPS, b *p* < 0.05;Compared with group Andro 6.25, c *p* < 0.05; Compared with group Andro 12.5, d *p* < 0.05. **A** PIIIP concentration. **B** TAT concentration. **C** ATIII concentration. **D** APC concentration. *ELISA* enzyme linked immunosorbent assay, *PIIIP* procollagen peptide type III, *TAT* thronbin-antithronbin complex, *ATIII* antithrombin III, *APC* activated protein C, *Andro* andrographolide

The secretions of thrombin antithrombin complex (TAT) and procollagen peptide type III (PIIIP) significantly were seen to increase but that of activated protein C (APC) and of antithrombin III (ATIII) to decrease from LPS stimulated RLE-6TN cells. Andro application, however, significantly reversed the changes of all factors above in dose-dependent manner. (Fig. 3).

### Andro Suppresses NF-κB Pathway Activation Following LPS Stimulation in RLE-6TN Cells

**Fig. 4:**
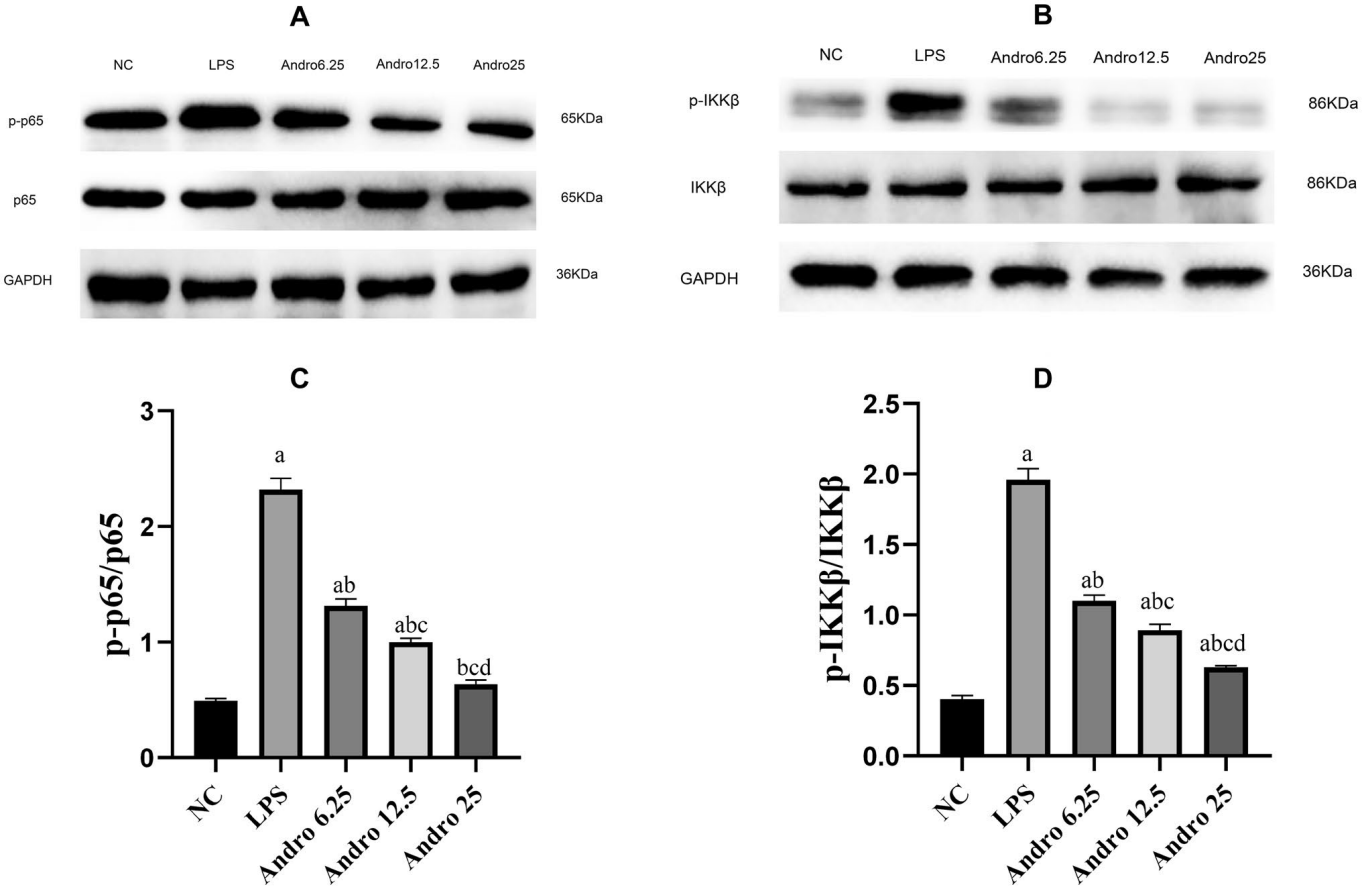
Effects of Andro on NF-κB signal pathway activation induced by LPS. Western blotting was performed to measure total p65, p-p65, total IKKβ and p-IKKβ protein expression in cells, GAPDH was used as an internal control for protein reference. Compared with group NC, a *p* < 0.05; Compared with group LPS, b *p* < 0.05; Compared with group Andro 6.25, c *p* < 0.05; Compared with group Andro 12.5, d *p* < 0.05. **A**, **B** Western-blot bands of p65, p-p65, total IKKβ and p-IKKβ respectively. **C** Relative protein expression of p-p65. **D** Relative protein expression of p-IKKβ. *p-p65* phosphorylated p65, *p-IKKβ* phosphorated IKKβ, *Andro* andrographolide

**Fig. 5:**
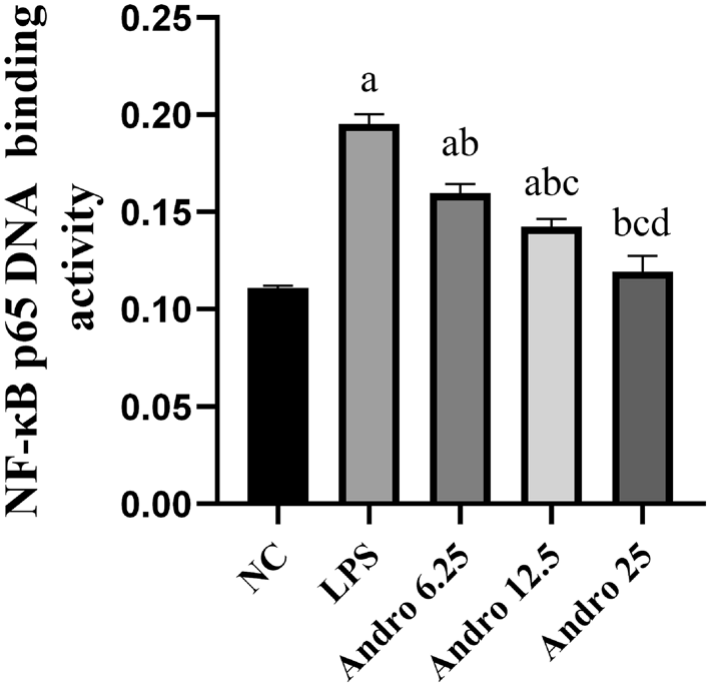
Effects of Andro on p65 DNA binding activity induced by LPS. ELISA was performed to measure the NF-κB p65 DNA binding in cell nucleus. Compared with group NC, a *p* < 0.05; Compared with group LPS, b *p* < 0.05; Compared with group Andro 6.25, c *p* < 0.05; Compared with group Andro 12.5, d *p* < 0.05. *Andro* andrographolide, *LPS* lipopolysaccharide

LPS stimulation caused significant activation of the NF-κB pathway in RLE-6TN cells, as shown by increased expressions of p-p65/p65 and p-IKKβ/IKKβ, as well as by the enhanced p65 DNA binding activity. Andro successfully inhibited p-p65/p65 and p-IKKβ/IKKβ expressions (Fig. 4) and weakened p65 DNA binding activity which meant NF-κB inactivation. (Fig. 5).

### p65 Gene Significantly Influences Andro's Effect on Coagulation and Fibrinolysis Inhibition-Related Factors in RLE-6TN Cells

**Fig. 6:**
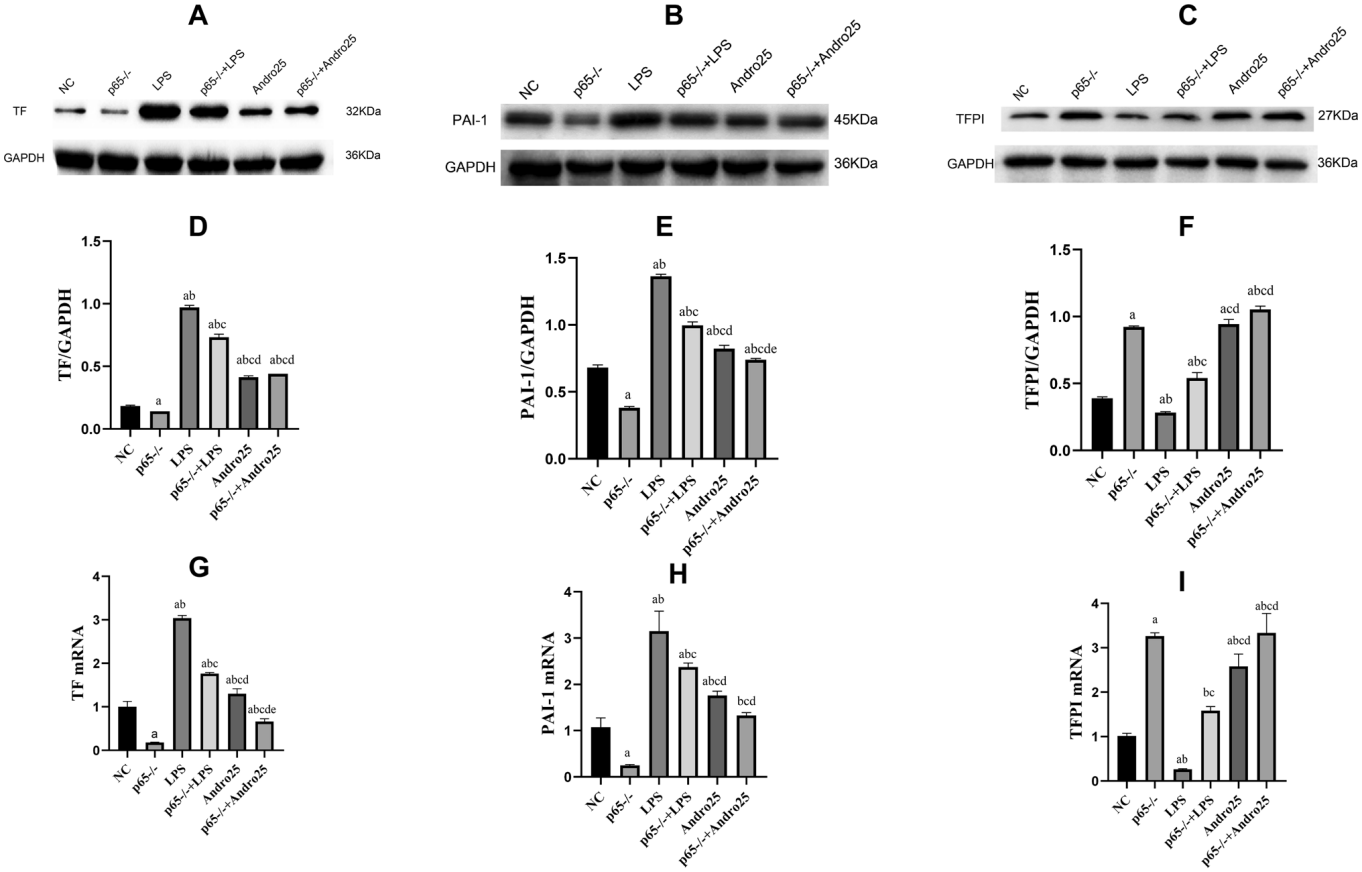
Effects of p65 gene down-regulation (p65^−/−^) with or without Andro on protein and mRNA expressions of TF, PAI-1 and TFPI in the cells. Western blotting and reverse transcription polymerase chain reaction were used to detect the protein and mRNA expressions of TF, PAI-1 and TFPI, respectively. GAPDH was used as an internal reference for protein. Compared with group NC, a *p* < 0.05; Compared with group p65^−/−^, b *p* < 0.05; Compared with group NC + LPS, c *p* < 0.05; Compared with group p65^−/−^ + LPS, d *p* < 0.05; Compared with group NC + Andro + LPS, e *p* < 0.05. **A** and **D** the protein expression of TF. B and E, the protein expression of PAI-1. **C** and **F** protein expression of TFPI. **G** TF mRNA expression. **H** PAI-1 mRNA expression. **I** TFPI mRNA expression. *TF* tissue factor, *TFPI* tissue factor pathway inhibitor, *PAI-1* plasminogen activator Inhibitor-1, *WB* western blot, *Andro* andrographolide

**Fig. 7:**
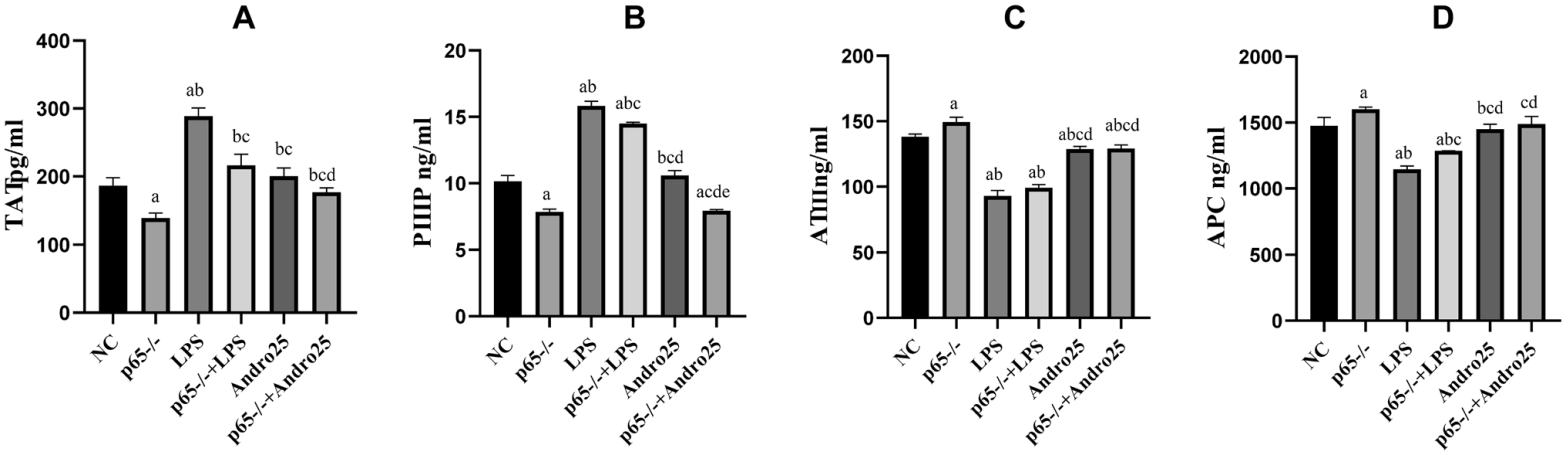
Effects of p65 gene down-regulation (p65^−/−^) with or without Andro on the secretions of TAT, PIIIP,ATIII and APC in the cells. ELISA was performed to measure the concentrations of PIIIP, TAT, ATIII and APC in supernatants. Compared with group NC, a *p* < 0.05; Compared with group p65^−/−^, b *p* < 0.05; Compared with group NC + LPS, c *p* < 0.05; Compared with group p65^−/−^ + LPS, d *p* < 0.05; Compared with group NC + Andro + LPS, e*p* < 0.05. **A** TAT concentration. **B** PIIIP concentration. **C** ATIII concentration. **D** APC concentration. *ELISA* enzyme linked immunosorbent assay, *TAT* thronbin–antithronbin complex, *IIIP* procollagen peptide type III, *ATIII* antithrombin III, *APC* activated protein C, *Andro* andrographolide

**Fig. 8:**
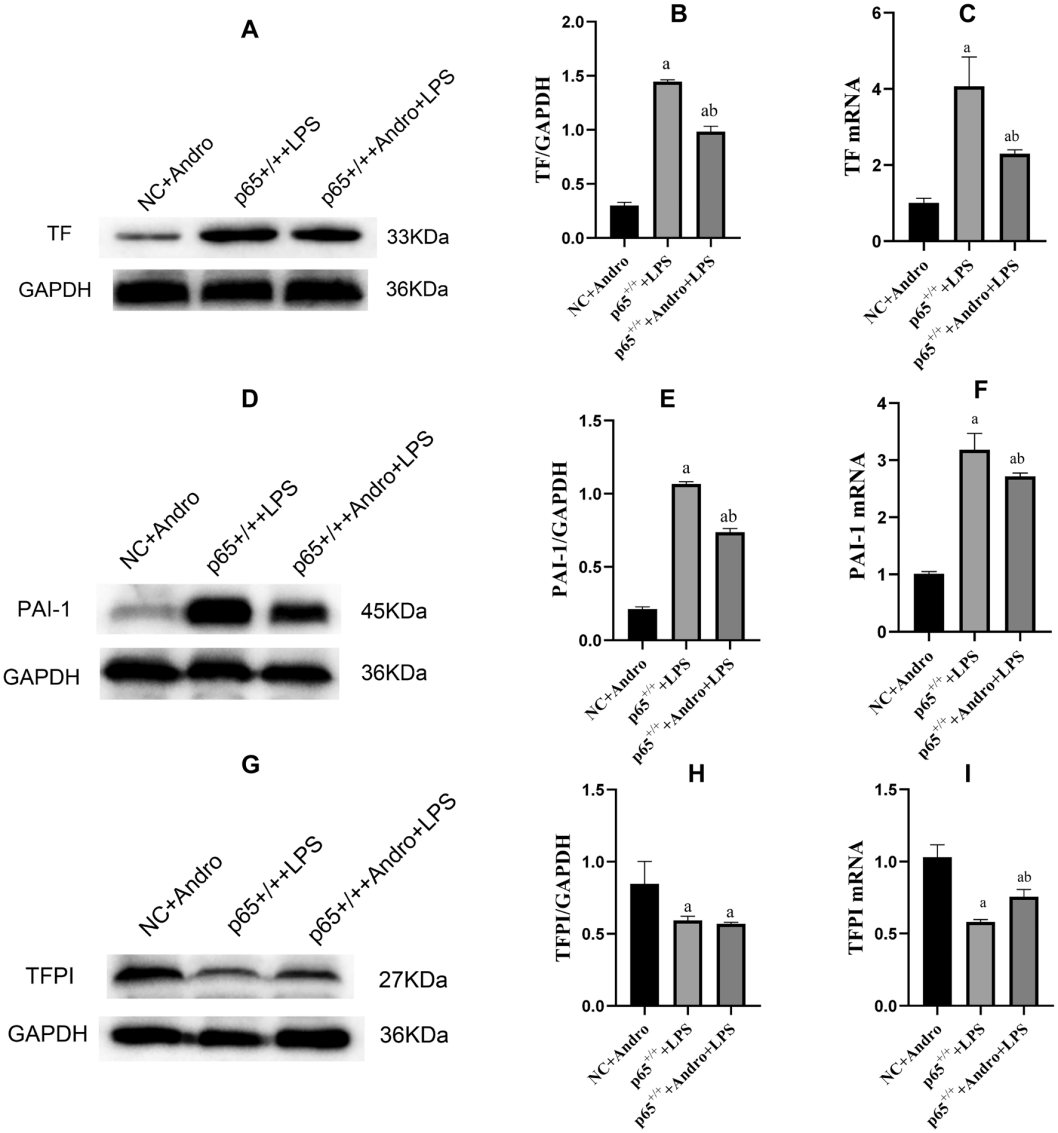
Effects of p65 gene up-regulation (p65^+/+^) with or without Andro on protein and mRNA expressions of TF, PAI-1 and TFPI in the cells. Western blotting and reverse transcription polymerase chain reaction were used to detect the protein and mRNA expressions of TF, PAI-1 and TFPI, respectively. GAPDH was used as an internal reference for protein. Compared with group NC + Andro, a *p* < 0.05; Compared with group p65^+/+^ + LPS, b *p* < 0.05; A and B, the protein expression of TF. **D** and **E** the protein expression of PAI-1. **G** and **H** protein expression of TFPI. **C** TF mRNA expression. **F** PAI-1 mRNA expression. **I** TFPI mRNA expression. *TF* tissue factor, *TFPI* tissue factor pathway inhibitor, *PAI-1* plasminogen activator inhibitor-1, *WB* western blot, *Andro* andrographolide

**Fig. 9:**
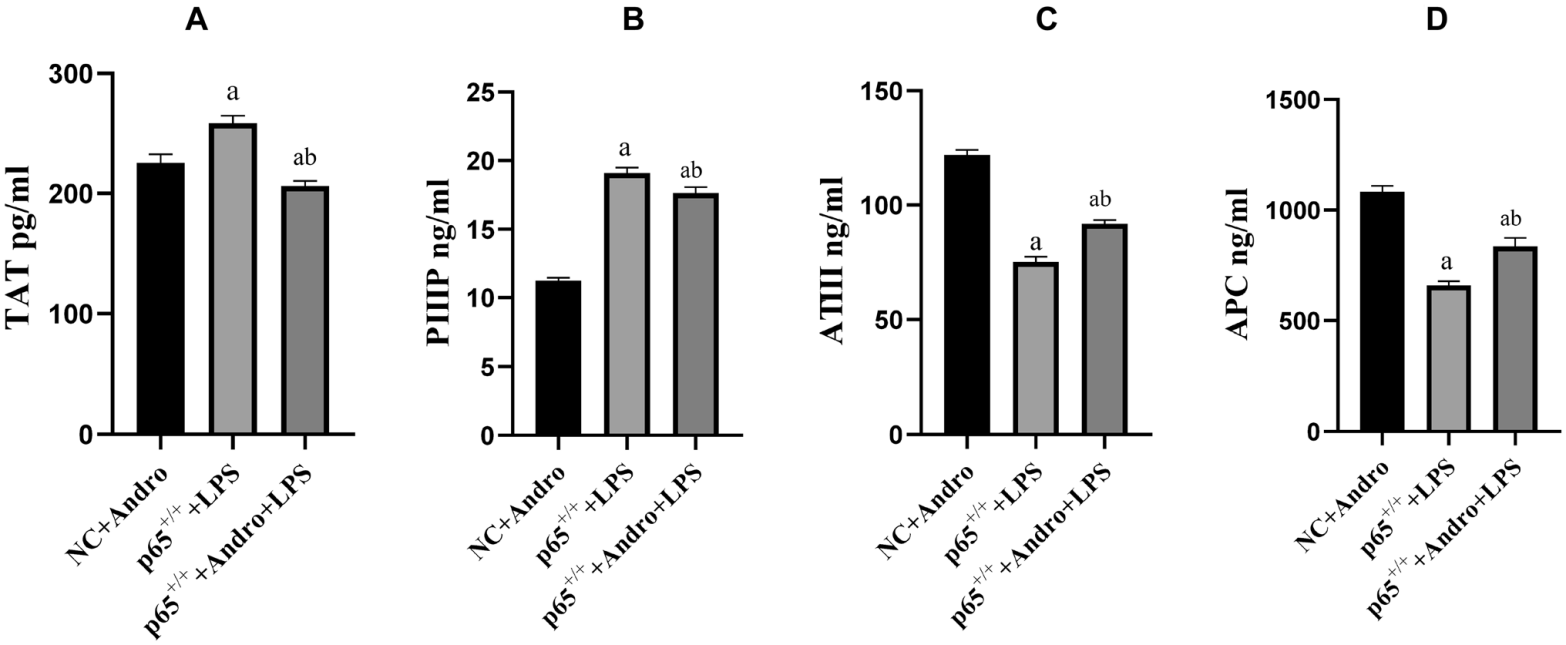
Effects of p65 gene up-regulation (p65^+/+^) with or without Andro on the secretions of TAT, PIIIP, ATIII and APC in the cells. ELISA was performed to measure the concentrations of TAT, PIIIP, ATIII and APC in supernatants. Compared with group NC + Andro, a p < 0.05; Compared with group p65^+/+^ + LPS, b p < 0.05; **A** TAT concentration. **B** PIIIP concentration. **C** ATIII concentration. **D** APC concentration. *ELISA* enzyme linked immunosorbent assay, *TAT* thrombin–antithronbin complex, *PIIIP* procollagen peptide type III, *ATIII* antithrombin III, *APC* activated protein C, *Andro* andrographolide

In order to further confirm whether the mechanism of Andro is associated with NF-κB pathway, we constructed cell lines with different p65 gene expressions. Our data interestingly showed that the efficacies of Andro on coagulation and fibrinolytic inhibition-related factors was tremendously enhanced with down-regulation of p65 (p65^−/−^) (Fig. 6 and Fig. 7) but was obviously weakened with up-regulation of p65 gene (p65^+/+^) (Fig. 8 and Fig. 9).

## Discussion

Alveolar epithelial cell type II (AECII) plays a pivotal role in the regulation of alveolar hypercoagulation and fibrinolytic inhibition in ARDS [10–12]. So we chose rat AECII cell line RLE-6TN as the experimental object. Gram negative bacterial infection such as bacteria pneumonia is the common cause of ARDS, and LPS is the most pathogenic factor of bacteria. So LPS is often used to replicate ARDS models in vitro and in vivo [19, 20]. According to our previous studies, 5 mg/l of LPS was selected to stimulate the cell [10, 11].

In ARDS process, alveolar hypercoagulation and fibrinolytic inhibition have been confirmed to be pivotal pathophysiologies, which mainly contribute to the refractory hypoxemia in ARDS. And our published data showed that alveolar epithelial cell type II (AEC II) essentially regulates coagulation and fibrinolytic inhibition by expressing coagulation and fibrinolytic inhibition related factors [10, 11], which was confirmed again in this study in vitro.

As described in our previous researches [7, 10], TF, PAI-1, TAT and PIIIP are associated with coagulation and fibrinolytic inhibition, while TFPI, APC and ATIII are anti-coagulators. So we selected these factors again as our observational targets. Our data showed that AEC域-mediated hypercoagulation and fibrinolytic inhibition happened in condition of LPS stimulation. However, Andro effectively reversed the changes of above coagulation and fibrinolytic-related factors in AECII cell induced by LPS stimulation, which demonstrated that Andro has obvious efficacies on procoagulation and fibrinolytic inhibition mediated by AECII cell under LPS provocation.

NF-κB pathway was confirmed to regulate alveolar hypercoagulation and fibrinolytic inhibition in ARDS in our research [7, 10]. Therefore, we explored whether this pathway is associated with the mechanism through which Andro exerts its efficacy on AECII cell mediated coagulation and fibrinolytic inhibition. Our results indicated that Andro significantly inhibited the LPS-stimulated NF-κB pathway activation, as shown by decreases of p-p65/p65 and p-IKKβ/IKKβ, and of p65 DNA binding activity. To further confirm the Andro’s mechanism, we control the p65 expression. Data indicated that Andro’s effects was obviously enhanced by down-regulation of p65 (p65^−/−^) but was remarkably weakened by up-regulation of p65 (p65^+/+^). So, we have the reason to think that Andro ameliorates the expressions of coagulation and fibrinolytic inhibition related factors and promotes productions of anti-coagulant factors through NF-κB pathway in LPS-treated AECII cell.

Interestingly, as happened in our animal study [18], the effects of Andro became more obvious when dosage of the drug from 6.25 to 25 µg/ml, suggesting a dose-dependent manner.

From the results of current cell experiment and previous animal study, it is worth to further explore the value of Andro in the future.

## Conclusion

Andrographolide ameliorates LPS induced expressions and secretions of procoagulant and fibrinolytic inhibitory factors in AECII through inactivation of NF signaling pathway. Our findings suggest the potential protective role of Andrographolide in alveolar hypercoagulation and fibrinolytic inhibition in ARDS.

## Data Availability

We could offer the data and material if there is any requirement.

## Abbreviations

ARDS: Acute respiratory distress syndrome
ALI: Acute lung injury
Andro: Andrographolide
TF: Tissue factor
TAT: Thrombin–antithrombin complex
PAI-1: Plasminogen activator inhibitor-1
PIIIP: Procollagen peptide type III
TFPI: Bronchoalveolar lavage fluid
APC: Activate protein C
AT-III: Antithrombin III
AECII: Alveolar epithelial cell type II
LPS: Lipopolysaccharide
ELISA: Enzyme-linked immunosorbent assay
FBS: Fetal bovine serum
RT-qPCR: Real-time fluorescence quantitative polymerase chain reaction
WB: Western blot
